# Characterization and phylogenetic analysis of the complete mitochondrial genome of the medicinal fungus *Laetiporus sulphureus*

**DOI:** 10.1038/s41598-018-27489-9

**Published:** 2018-06-14

**Authors:** Qiang Li, Mei Yang, Cheng Chen, Chuan Xiong, Xin Jin, Zhigang Pu, Wenli Huang

**Affiliations:** 1 Biotechnology and Nuclear Technology Research Institute, Sichuan Academy of Agricultural Sciences, Chengdu, 610061 Sichuan P.R. China; 2 Panzhihua City Academy of Agricultural and Forest Sciences, Panzhihua, 617061 Sichuan P.R. China; 3 Key Laboratory of Bio-Resource and Eco-Environment of Ministry of Education, College of Life Sciences, Sichuan University, Chengdu, 610065 Sichuan P.R. China; 4 Institute of plant protection, Sichuan Academy of Agricultural Sciences, Chengdu, 610066 Sichuan P.R. China; 5 Present Address: Sichuan Academy of Agricultural Sciences, 106 # Shizishan Rd, Chengdu, 610061 Sichuan, China

## Abstract

The medicinal fungus *Laetiporus sulphureus* is widely distributed worldwide. To screen for molecular markers potentially useful for phylogenetic analyses of this species and related species, the mitochondrial genome of *L. sulphureus* was sequenced and assembled. The complete circular mitochondrial genome was 101,111 bp long, and contained 38 protein-coding genes (PCGs), 2 rRNA genes, and 25 tRNA genes. Our BLAST search aligned about 6.1 kb between the mitochondrial and nuclear genomes of *L. sulphureus*, indicative of possible gene transfer events. Both the GC and AT skews in the *L. sulphureus* mitogenome were negative, in contrast to the other seven *Polyporales* species tested. Of the 15 PCGs conserved across the seven species of *Polyporales*, the lengths of 11 were unique in the *L. sulphureus* mitogenome. The Ka/Ks of these 15 PCGs were all less than 1, indicating that PCGs were subject to purifying selection. Our phylogenetic analysis showed that three single genes (*cox1, cob*, and *rnl*) were potentially useful as molecular markers. This study is the first publication of a mitochondrial genome in the family *Laetiporaceae*, and will facilitate the study of population genetics and evolution in *L. sulphureus* and other species in this family.

## Introduction

The fruiting body of *Laetiporus sulphureus* (Bull.) Murill, 1904 (*Basidiomycota*: *Polyporales*), with its striking citrus-yellow to pale orange color, is considered a cosmopolitan species, distributed from the boreal to tropical climactic zones^1^. *L. sulphureus* is found on a variety of substrates, including the decaying logs, stumps, and trunks of coniferous and deciduous trees^1^. *L. sulphureus* is a traditional food^1–4^, which produces a variety of bioactive compounds, with a variety of medicinal effects^5,6^. Several polysaccharides, triterpenoids, triterpenes, and sesquiterpenoids that have been extracted from the fruiting bodies and mycelium of *L. sulphureus* have potential antitumoral, antioxidant, and antimicrobial effects^7–11^. To date, various authors have described up to 12 species in *Laetiporus*; *L. sulphureus* has been designated the type species of this genus^12–15^. *Laetiporus* species vary substantially, and can be distinguished by basidiocarp color, by host range, and by internal transcribed spacer (ITS) sequence^13,15^.

Mitochondria generate most of the chemical energy used by the cell^16,17^. Each mitochondria contains its own genome, an indication that mitochondria were originally derived from bacteria through endosymbiosis^18,19^. Over evolutionary time, the mitochondrial genomes of different eukaryotes have been greatly altered with respect to genome size, gene content, and gene order^20,21^. With the development of next-generation sequencing technology, an increasing number of mitochondrial genomes have been sequenced^22,23^. Mitochondrial genomes have several advantages over nuclear genomes, including their relatively small size, limited rate of recombination, evolution independent of nuclear genes, and abundant homologous genes; these advantages render mitochondrial genomes powerful tools with which to investigate population genetics and evolutionary biology^24,25^. However, unlike the mitogenomes of animals and plants, fungal mitogenomes are little understood, especially in *Basidiomycetes*^26,27^. Mitochondria are important in the growth and development of fungi; they are critically involved in fungal senescence, quiescence, virulence, pathogenicity, and drug resistance^28,29^. The mitochondrial genomes of fungal species vary remarkably in size, gene order, and number of repeats, intergenic regions, introns, and open reading frames (ORFs)^30–32^.

To date, seven mitochondrial genomes in the order *Polyporales* have been published: *Ganoderma applanatum*^27^, *G. lucidum*^33^, *G. meredithae*^34^, *G. sinense* (GenBank: KF673550), *Phlebia radiate*^35^, *Trametes cingulate*^36^, and *Fomitopsis palustris* (GenBank: AP017926). These mitochondrial genomes increase our understanding of the evolution and differentiation of mitochondrial genomes in *Polyporales*, and also provide evidence for phylogenetic relationships among different species. *L. sulphureus* and related species are widely distributed worldwide^4,15^ (across Europe, Africa, South America, and Asia). The wide distribution of this species, and the large number of populations involved, have made the characterization of genetic variation and phylogenetic relationships within *Laetiporus* difficult^12,13,15^. Indeed, no complete mitochondrial genome has been published within the genus *Laetiporus* and even the family *Laetiporaceae*. This information gap severely limits our understanding of the phylogenetic relationships within *Laetiporaceae* and the evolutionary history of this family. To date, the only available DNA bar code sequences for *L. sulphureus* identification are the ribosomal sequences for *ITS*, the large subunit (*LSU*), and the small subunit (*SSU*)^12,14^. Because few molecular markers are available with which to analyze the phylogenetic relationships and genetic variation within *Laetiporus*, our understanding of the population genetics of *L. sulphureus* is limited.

Here, we assembled and annotated the complete mitochondrial genome of *L. sulphureus*, and determined genome content and organization. We performed a comparative mitogenomic analysis of seven fungal species in *Polyporales* to identify regions of variation and conservation across genomes, particularly with respect to genome organization, gene content, and gene order. We also analyzed the phylogenetic relationships within *Agaricomycetes* based on mitochondrial genomes. To the best of our knowledge, this is the first mitochondrial genome reported in the *Laetiporaceae*.

## Results

### General features of the mitogenome of *L. sulphureus*

**Figure 1:**
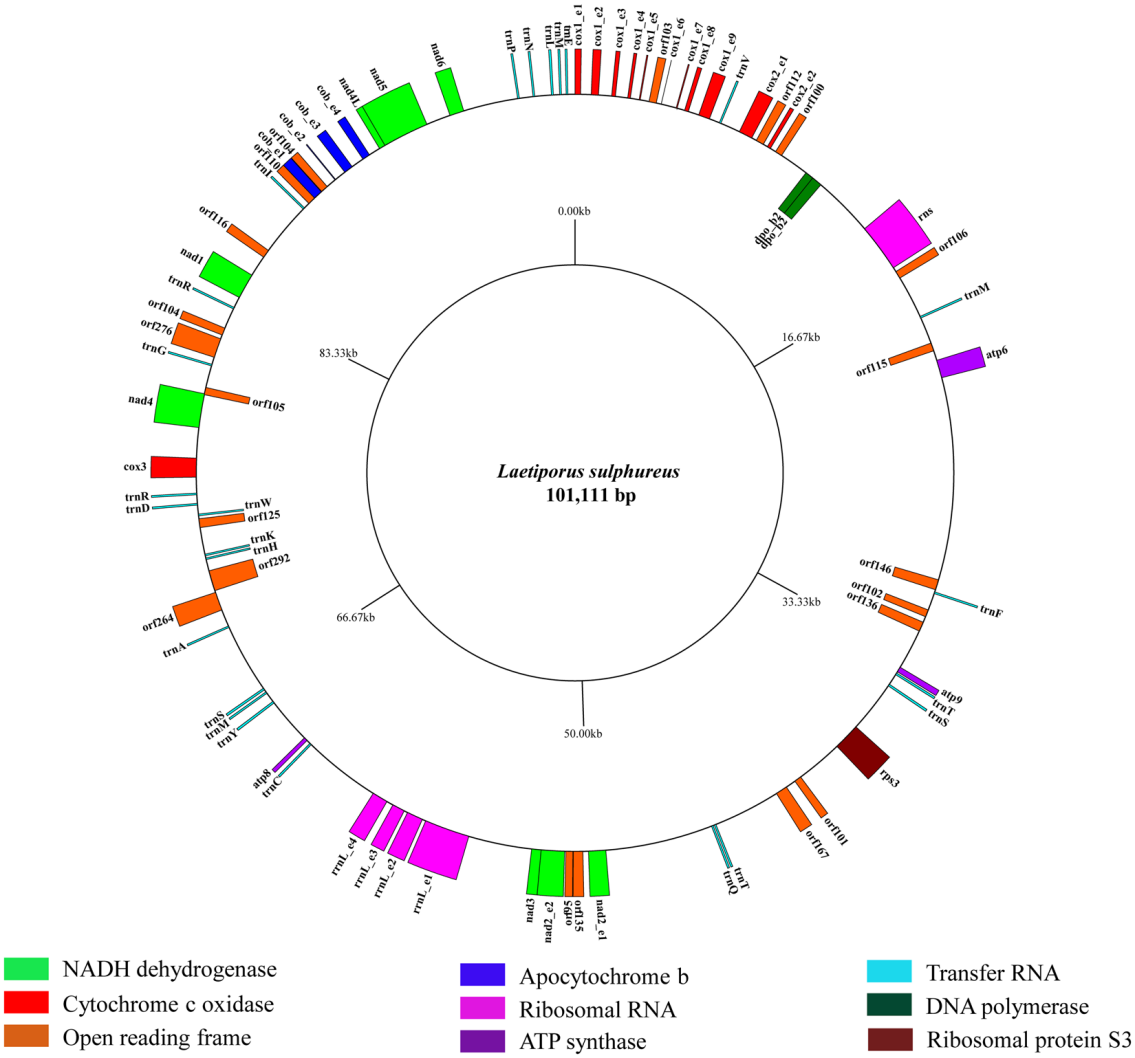
Circular map of the *Laetiporus sulphureus* mitochondrial genome. Various genes are represented with different color blocks. The color blocks outside the ring indicate genes that are located on the direct strand, and color blocks within the ring indicate genes that are located on the reverse strand.

The mitogenome of *L. sulphureus* was 101,111 bp long and was circular (Fig. 1). The complete mitogenome contained 38 PCGs, 25 tRNA genes, and 2 rRNA genes (Table S1). Of the 38 PCGs found in the *L. sulphureus* mitogenome, 14 were conserved core protein-coding genes (*atp6, atp8, atp9, cox1, cox2, cox3, cob, nad1, nad2, nad3, nad4, nad4L, nad5*, and *nad6*), one was a ribosomal protein S3 gene (*rps3*), four were intronic ORFs (orf103, orf112, orf135, and orf104) with homing endonuclease domains, two were ORFs similar to DNA polymerase type B genes, and the remaining 17 were ORFs with unknown function. The *cox1* gene contained the most number of introns in the *L. sulphureus* mitogenome. IB was the most common intron types (Table S1). Of the 65 total genes, 53 genes were located on the J strand, and 12 (9 PCGs and 3 tRNAs) were located on the N strand (Fig. 1).

The *L. sulphureus* mitogenome contained two overlapping nucleotides that were located in the neighboring genes *nad4*/orf105 (−38 bp) and nad4L/nad5 (−1 bp). We detected 62,744 intergenic nucleotides in the *L. sulphureus* mitogenome, each ranging from 2 to 8,694 bp long. The longest intergenic region was located between *atp6* and orf146, indicating that the *L. sulphureus* mitogenome had a relaxed structure (Table S1).

**Figure 2:**
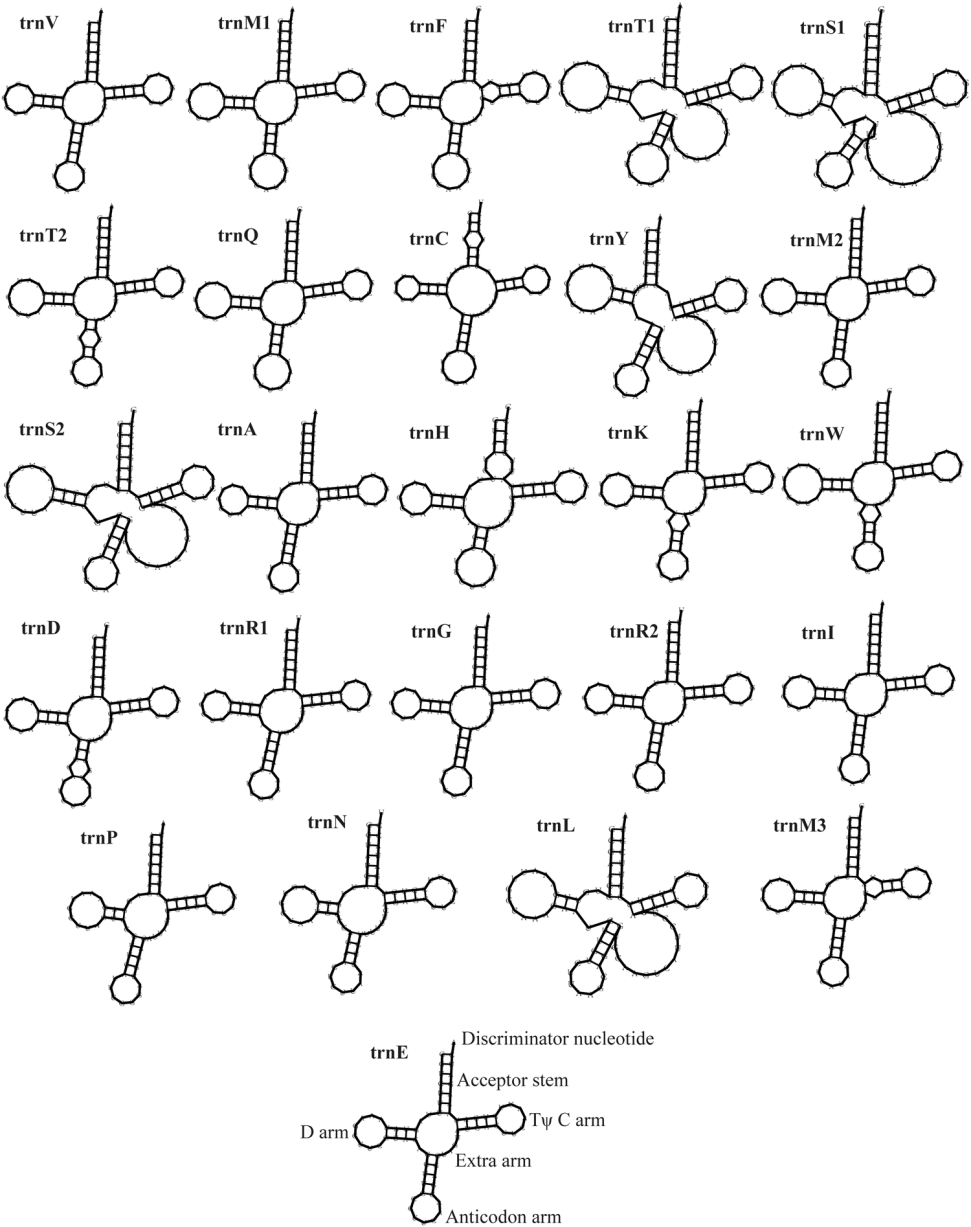
Putative secondary structures of the 25 tRNA genes identified in the mitochondrial genome of *Laetiporus sulphureus*. All tRNA genes are shown in the order of occurrence in the mitochondrial genome, starting from trnV.

**Figure 3:**
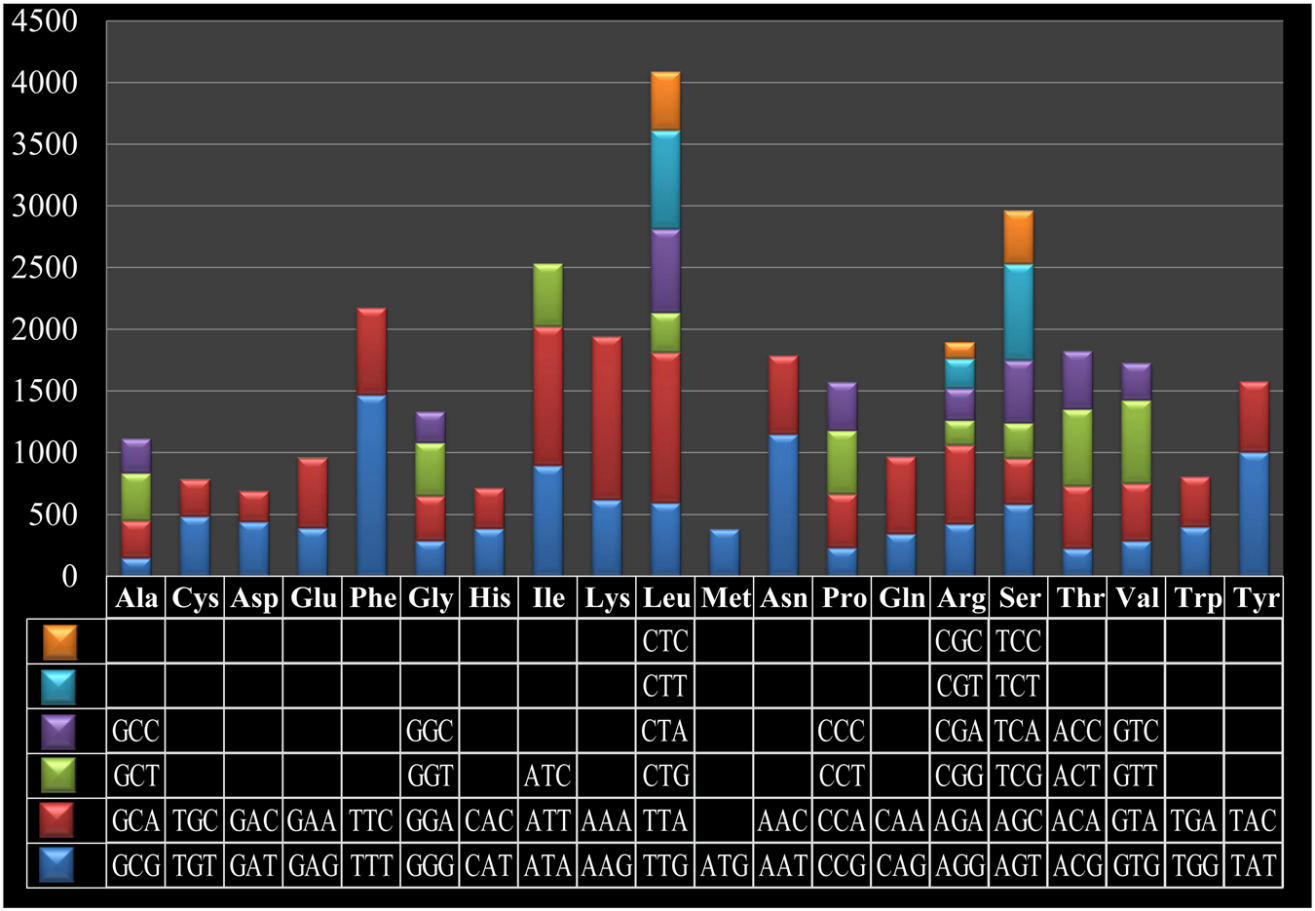
Codon usage in the *Laetiporus sulphureus* mitochondrial genome. Frequency of codon usage is plotted on the y-axis.

*L. sulphureus* contained 25 tRNAs, which are folded into a classical cloverleaf secondary structure (Fig. 2). These tRNAs encode for 20 standard amino acids. The length of each tRNA varied, ranging from 71 to 86 bp, mainly due to changes in the size of the extra arm (Fig. 2). Two tRNA genes with different anticodons coded for the amino acids threonine, arginine, and serine; methionine was coded for by three tRNAs with the same anticodons. Analysis of codon usage indicated that ATT, AAA, and TTA are the most frequent codons for isoleucine (Ile), lycine (Lys), and leucine (Leu), respectively (Fig. 3), which contribute to the high AT content of the *L. sulphureus* mitogenome (63.7%). Although codons containing A or T, including AAA, TTT, TTA, AAT, ATT, TAT and ATA, were the most frequently used codons across all of the *Polyporales* mitogenomes examined, these codons are used less frequently in the mitogenome of *L. sulphureus* than in other species of *Polyporales* (Table S2). The frequencies of codons containing G or C (e.g. CTT, TCT, GTT, TTC, AAC and ACT) were significantly higher in the *L. sulphureus* mitogenome than in the mitogenomes of other species of *Polyporales*, indicating that the GC content in the *L. sulphureus* mitogenome (36.3%) was greater than in the other species of *Polyporales* tested (24.0–31.2%).

### Repetitive elements in the *L. sulphureus* mitogenome

BLASTn searches of the *L. sulphureus* mitogenome against itself identified 36 repeat regions (Table S3). The size of the repeats ranged from 30 to 152 bp; repeat sequence similarity was between 86.28% and 100%. The largest repeat region in the *L. sulphureus* mitogenome was 152 bp. This region was located in the intergenic region between *atp6*/orf146 and the protein coding region orf276. Repeat sequences accounted for 2.14% of the entire *L. sulphureus* mitogenome.

We detected 15 tandem repeats in the *L. sulphureus* mitogenome (Table S4). The length of the longest tandem sequence was 77 bp, with two copies. Across all tandem repeats, most had either two or four copies. Tandem repeat sequences accounted for 0.65% of the entire mitochondrial genome. We identified 35 forward, 10 palindromic, and 5 reverse repeats, accounting for 2.93% of the entire mitogenome (Table S5).

To identify gene segments had been transferred between the nuclear and mitochondrial genomes, we blasted nuclear genomic sequences against the *L. sulphureus* mitogenome. We found 45 aligned fragments, 29–654 bp long, with sequence identities between 82.65% and 100% (Table S6). The largest aligned fragment was found encompassed the protein coding regions orf102 and orf136, as well as the intergenic region between them. The presence of large fragments that aligned between the nuclear and mitochondrial genomes of *L. sulphureus* indicated that genetic transfer between mitochondrial and nuclear genome may have occurred over evolutionary time.

### Gene variability in mitogenome

The mitogenome of *L. sulphureus* was larger than the mitogenomes of *F. palustris*, *G. lucidum*, *G. meredithae*, *G. sinense*, and *T. cingulate*, but smaller than the mitogenomes of *G. applanatum*, and *P. radiata* (Table 1). The number of introns and tRNA genes in these mitogenomic sequences varied. The mitogenome of *P. radiate* contained the most introns, while that of *G. meredithae* contained the most tRNA genes. In the mitogenome of *L. sulphureus*, both AT skew and GC skew were negative, whereas the AT skew and GC skew of all other *Polyporales* mitogenomes examined were positive (Table 1). This result suggested that the nucleotide composition of the *L. sulphureus* mitogenome was more varied than that of other *Polyporales* species.

**Table 1:** Comparison of *Polyporales* mitogenomes.

	*Ganoderma applanatum*	*Ganoderma lucidum*	*Ganoderma meredithae*	*Ganoderma sinense*	*Phlebia* *radiata*	*Trametes cingulata*	*Fomitopsis palustris*	*Laetiporus sulphureus*
Phylum	*Basidiomycota*	*Basidiomycota*	*Basidiomycota*	*Basidiomycota*	*Basidiomycota*	*Basidiomycota*	*Basidiomycota*	*Basidiomycota*
Order	*Polyporales*	*Polyporales*	*Polyporales*	*Polyporales*	*Polyporales*	*Polyporales*	*Polyporales*	*Polyporales*
Family	*Polyporaceae*	*Polyporaceae*	*Polyporaceae*	*Polyporaceae*	*Meruliaceae*	*Polyporaceae*	*Fomitopsidaceae*	*Laetiporaceae*
Accession number	KR109212	KC763799	KP410262	KF673550	HE613568	GU723273	AP017926	MG519331
Genome size (bp)	119803	60635	78447	86451	156348	91500	63479	101111
GC content (%)	26.7	26.7	26.1	26.8	31.2	24.5	24.0%	36.3
AT-skew	0.019	0.002	0.003	0.031	0.003	0.001	0.013	−0.026
GC-skew	0.046	0.059	0.041	0.066	0.016	0.091	0.016	−0.052
No. of introns	29	11	18	30	36	26	6	16
No. of tRNAs	25	26	29	28	28	25	26	25

**Figure 4:**
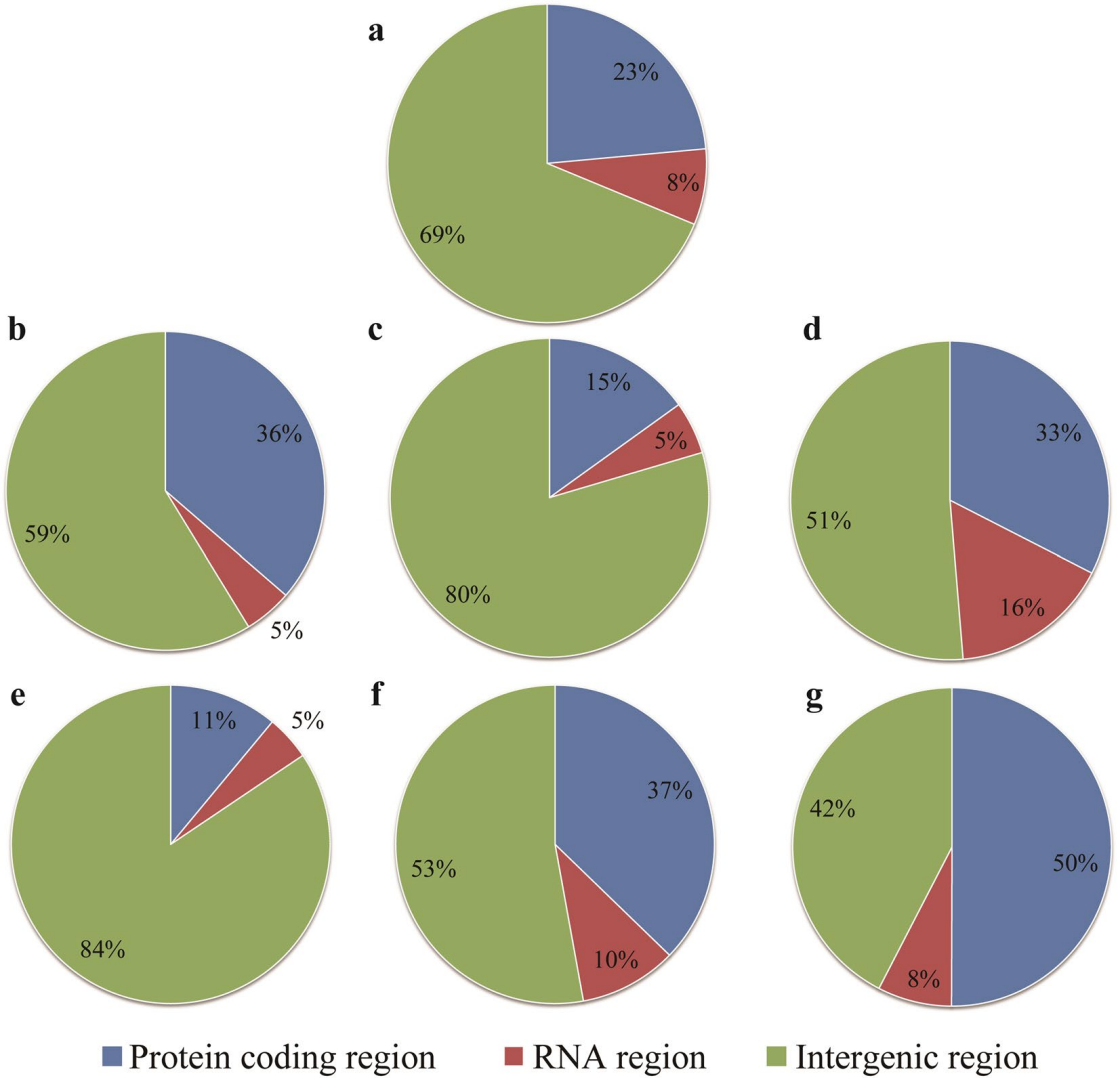
The intergenic, protein-coding, and RNA gene regions as proportions of the entire mitochondrial genomes of seven *Polyporales* species: (**a**) *Laetiporus sulphureus*; (**b**) *Phlebia radiate*; (**c**) *Trametes cingulate*; (**d**) *Ganoderma lucidum*; (**e**) *G. applanatum*; (**f**) *G. meredithae*; (**g**) *G. sinense*.

Intergenic regions accounted for the largest part of the *L. sulphureus* mitogenome (69%), followed by protein-coding regions (23%; Fig. 4). RNA sequences (tRNA and rRNA) accounted for only 5% to 16% of the *Polyporales* mitogenomes. *G. applanatum* contained the highest proportion of intergenic regions, up to 84%, suggesting that this species may have the most relaxed mitochondrial structure of all *Polyporales* species investigated. Across the *Polyporales* mitogenomes investigated, the total length of the intergenic sequences was always longer than the total length of the protein-coding sequences, with an exception of the *G. sinense* mitogenome. In the *G. sinense* mitogenome, protein-coding sequences accounted for 50% of the total mitogenomic length. Changes in mitogenomic size were not always closely related to changes in the proportion of individual region types, as observed in *Polyporales*: the size of the mitogenome was affected by changes in the sizes of both the protein-coding and the intergenic regions.

### Gene rearrangements

**Figure 5:**
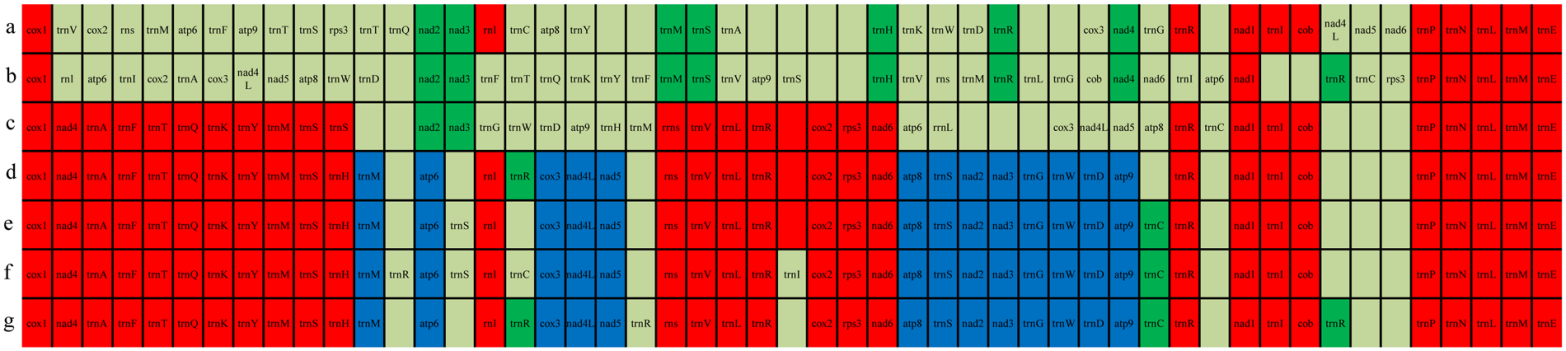
Comparison of gene order across seven *Polyporales* mitogenomes. Genes conserved across at least 5 species are shown in red; genes conserved across four species are shown in blue; genes conserved across two or three species are shown in green. (**a**) *Laetiporus sulphureus*; (**b**) *Phlebia radiate*; (**c**) *Trametes cingulate*; (**d**) *Ganoderma lucidum*; (**e**) *G. applanatum*, (**f**) *G. meredithae*; (**g**) *G. sinense*.

Gene order and rearrangement events in the mitochondrial genome might be informative for fungal evolution^37,38^. The order of mitochondrial genes from *trnP* to *cox1* is conserved across the mitogenomes of *Polyporales* species (Fig. 5). The order of most mitochondrial genes among the different species of *Ganoderma* analyzed was identical, indicating a close phylogenetic relationship among these species. Based on relative gene order, *T. cingulate* might cluster with *Ganoderma* species; the mitogenomes of *T. cingulate* and all species of *Ganoderma* analyzed here had 27 genes in identical positions. However, substantial variations in gene order were observed in *L. sulphureus* and *P. radiate*, indicating that gene rearrangements may have occurred several times during the evolution of these two species.

**Figure 6:**
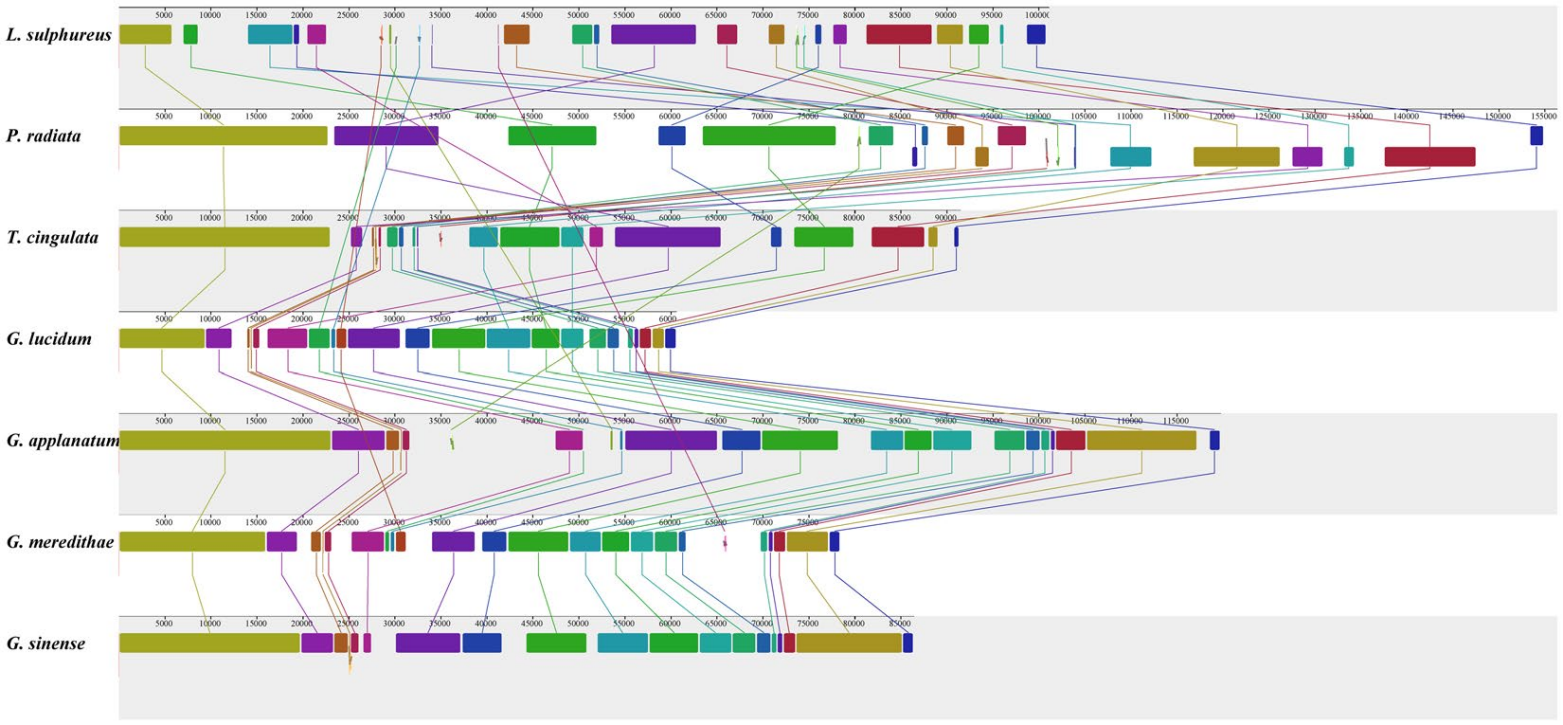
Comparative mitogenomic gene rearrangement analysis of seven *Polyporales* species using Mauve 2.4.0. We detected 26 homologous fragments in the mitochondrial genome across seven species in order *Polyporales*. The sizes and relative positions of homologous fragments varied among species.

Comparative genomic analysis also indicated that many gene rearrangements in the mitogenomes of the *Polyporales* species had occurred (Fig. 6). The mitochondrial genomes of *Polyporales* were divided into 26 homologous clusters; the size and relative position of these homologous clusters differed substantially among the species we investigated. Some homologous clusters may have been lost over evolutionary time, leading to decreased mitogenomic length. Based on the arrangement of homologous clusters, there was a high degree of synteny among the *Ganoderma* species. However, gene rearrangements were frequently observed in the mitogenomes of other species in order *Polyporales*, suggesting that gene order is highly variable at different taxonomic levels.

### Evolutionary rates of common genes in *Polyporales*

**Figure 7:**
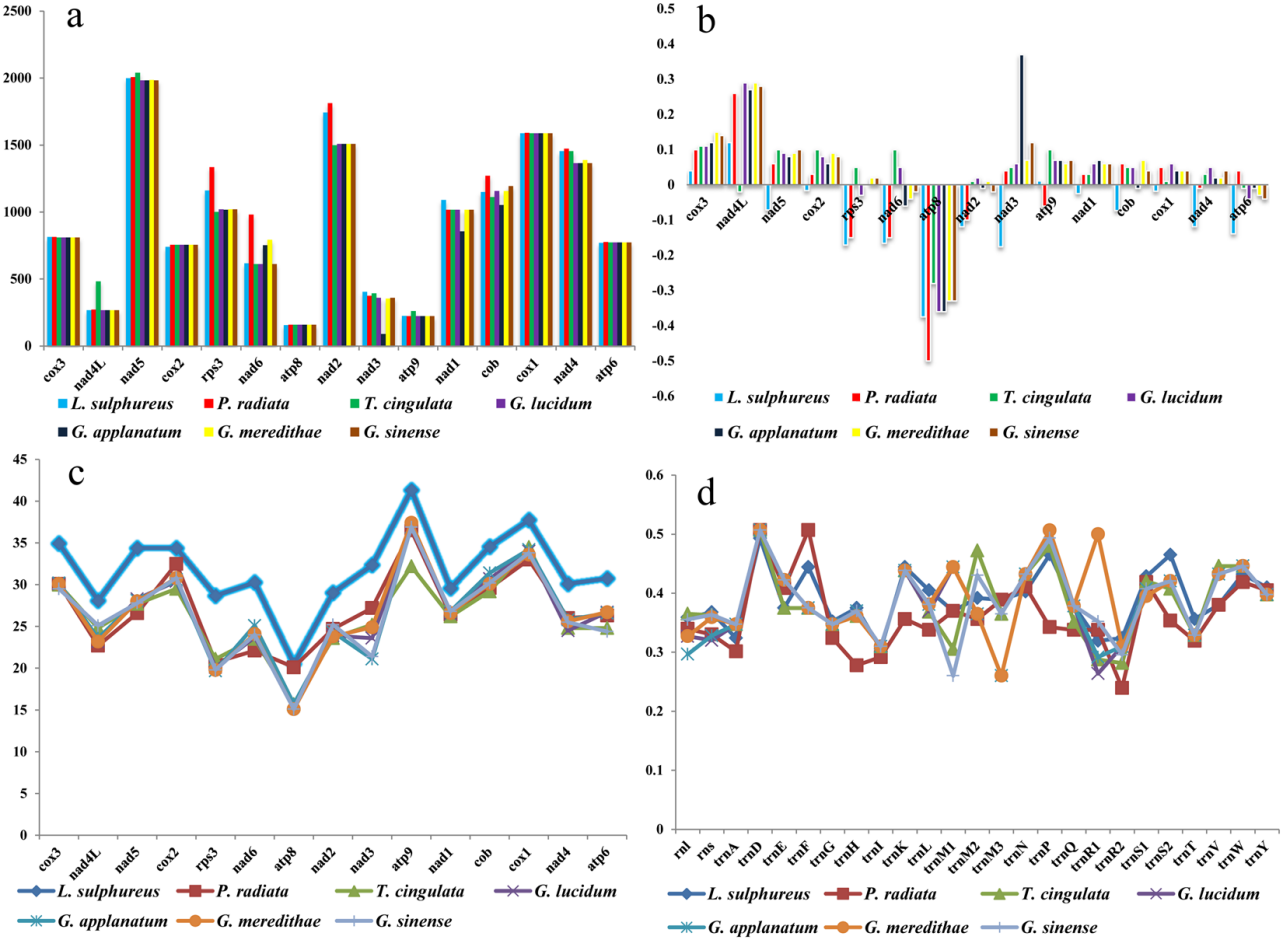
Variations in length and base composition of 15 protein-coding genes (PCGs) in seven mitochondrial genomes in order *Polyporales*. (**a**) length variation; (**b**) GC skew; (**c**) GC content across all 15 genes; (**d**) GC content of shared RNA genes only (tRNA and rRNA).

The lengths of the 14 conserved PCGs as well as that of the *rps3* gene varied across the mitogenomes examined (Fig. 7a). In *L. sulphureus*, the lengths of the genes *nad5, cox2, rps3, nad6, atp8, nad2, nad3, atp9, nad1, cob*, and *atp6* were unique across all mitogenomes examined. There was less length variation among the mitogenomes of the species of *Ganoderma*. Most of the PCGs in the *L. sulphureus* mitogenome had negative GC skews (*nad5, cox2, nad3, nad1* and *cox1*), but the GC skews of *cox3*, *atp9*, and *nad4L* were positive (Fig. 7b). This result is inconsistent with the other *Polyporales* species tested. The GC content of the *L. sulphureus* mitogenome was the highest among all the 7 *Polyporales* species tested, up to 36.3%, possible resulting from the relatively high GC content of protein encoding genes in *L. sulphureus* mitogenome (Fig. 7c). The GC content of the RNA genes did not vary greatly across the species tested (Fig. 7d). Therefore, the gene content of the *L. sulphureus* mitogenome was highly variable compared to that of other species of *Polyporales*.

**Figure 8:**
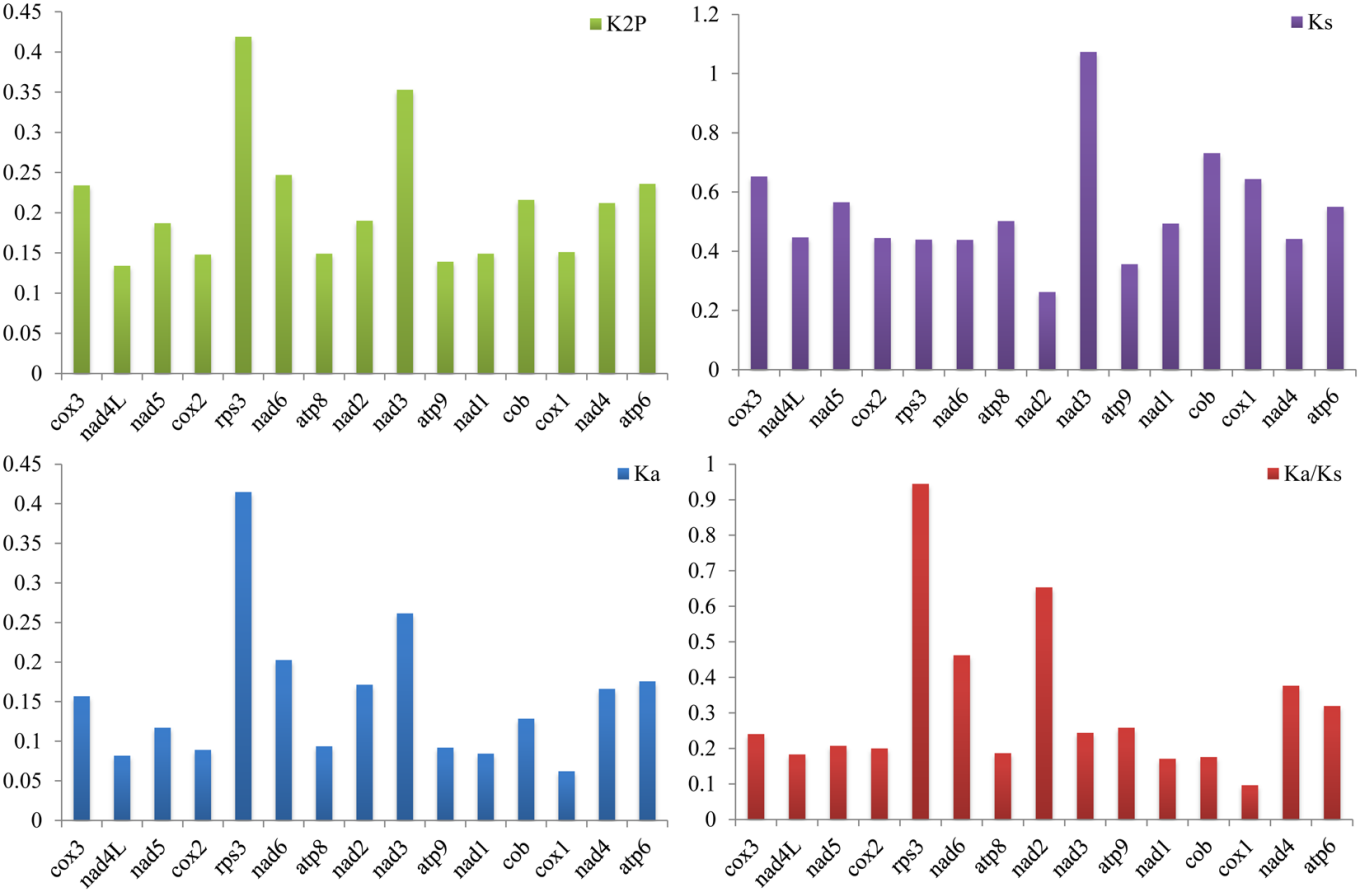
Genetic analysis of 15 protein coding genes of seven *Polyporales* mitochondrial genomes. K2P, the Kimura-2- parameter distance; Ka, the number of nonsynonymous substitutions per nonsynonymous site; Ks, the number of synonymous substitutions per synonymous site.

Different PCGs had different substitution rates in the different species investigated. Across all 15 PCGs detected, *rps3* contained the largest genetic distances among the seven *Polyporales* species, followed by *nad3* and *atp6* (Fig. 8). *nad4L* had the smallest genetic distance among different species, indicating that it was highly conserved. The synonymous substitution rate (Ks) of *nad3* was the highest across all the mitogenomes we examined, while that of *nad2* was the lowest. *rps3* and *nad3* had relatively high nonsynonymous substitution rates (Ka) at the nucleotide and amino acid levels. The Ka/Ks values for all 15 PCGs were <1, suggesting that these genes continue to evolve under purifying selection.

### Phylogenetic analysis

**Figure 9:**
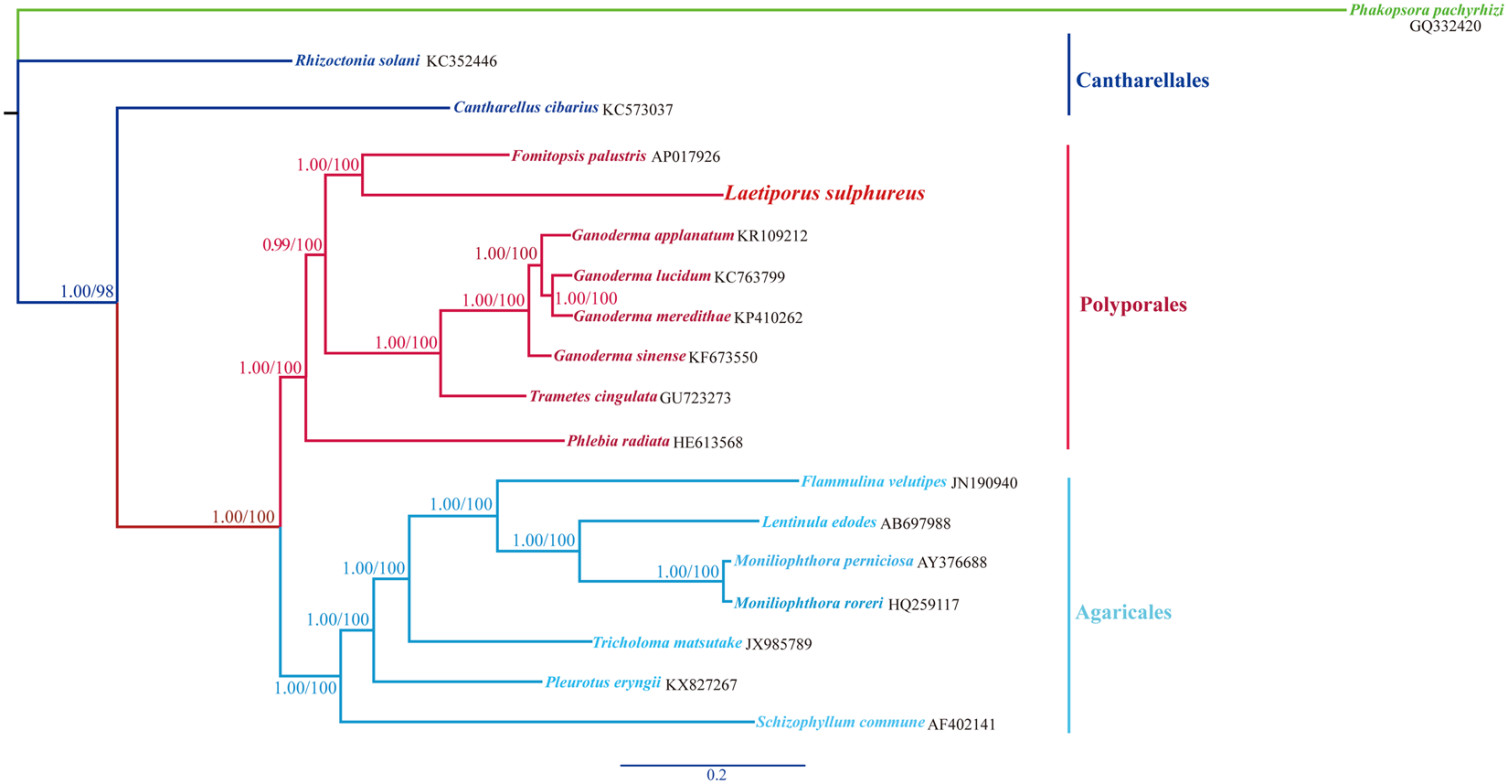
Molecular phylogeny of 18 *Agaricomycetes* species based on Bayesian inference and maximum likelihood analysis of 15 concatenated protein-coding genes. *Phakopsora pachyrhizi* was used as an outgroup. Support values are Bayesian posterior probabilities (before slash) and bootstrap values (after slash). IDs after species names are GenBank accession numbers; *Laetiporus sulphureus* was sequenced in this study.

Our ML and BI phylogenies of the *Agaricomycetes* species were congruent (Fig. 9). All recovered clades were well supported (Bootstrap (BS) ≥ 98; Bayesian posterior probability (BPP) = 1.00). *L. sulphureus* formed a clade with *F. palustris*, *G. applanatum*, *G. lucidum*, *G. meredithae*, *G. sinense*, *P. radiata* and *T. cingulate*, corresponding to the *Polyporales*; there was a sister relationship between *L. sulphureus* and *F. palustris* (BPP = 1.00; BS = 100). *T. cingulate* fell within *Ganoderma*, consistent with the results of the gene rearrangement analysis.

Single-gene tree topologies varied (Fig. S1), indicating incongruent phylogenetic signals among different genes. However, the BI phylogenies based on three genes (*cox1, cob*, and *rnl*) were consistent with the all-PCG phylogeny. Therefore, these genes might be useful barcode sequences for species identification and phylogenetic analysis within the *Agaricomycetes*.

## Discussion

The lengths of mitochondrial genomes in *Polyporales* varied, ranging from 60,635 to 156,348 bp. Previous studies have shown that mitogenomic length variations were primarily due to the size and number of introns, the frequency of duplicated repeats, and the presence of new genes introduced by horizontal transfer^39^. Here, the *P. radiata* mitogenome was the longest of all *Polyporales* species tested, and had the most introns^35^. However, the length of the mitogenome was not always positively correlated with the number of introns. For example, the *L. sulphureus* mitogenome contained fewer introns than did *G. sinense*, but the *L. sulphureus* mitogenome was much longer. Protein-coding and intergenic regions also contributed to length variations in the mitochondrial genomes we studied.

Out of the 38 complete *Basidiomycetes* mitochondrial genomes previously published (https://www.ncbi.nlm.nih.gov/genome/browse/?report=5), the GC content of the *L. sulphureus* mitogenome was higher than any other species tested, except for *Rhizoctonia solani*^40^. This suggested that there has been much GC content variation over the course of the mitochondrial evolution of *L. sulphureus*. GC and AT skews may be related to selection pressure on different bases in the genome^41^. The GC skew has been shown to indicate the leading strand, lagging strand, replication origin, and replication terminus^41^. Here, both AT and GC skews were negative in the *L. sulphureus* mitogenome, in contrast to the skews calculated for other species of *Polyporales*, indicating that the *L. sulphureus* mitogenome was subject to different selection pressures than were the mitogenomes of other *Polyporales* species.

The number of tRNA genes in the *Polyporales* mitogenomes ranged from 25 to 29, encoding for all 20 standard amino acids. Arginine, methionine, and serine in the *Polyporales* mitogenomes were all coded for by at least two tRNAs. However, only in the *L. sulphureus* mitogenome did two *trnT*s encode for threonine, indicating that *trnT* has been duplicated over the evolution of *L. sulphureus*. Our secondary structure analysis suggested that all tRNAs in the *L. sulphureus* mitogenome were folded into a classical cloverleaf structure, but the size of each tRNA varied, ranging from 71 to 86 bp. We found that this length variation was due to the large extra arms of *trnT*, *trnS*, *trnY*, and *trnL*. It was found that, in tRNAs, many mismatches and mutations occur on the anticodon arm (TψC), and on the acceptor stem. Mutations in the *L. sulphureus* mitogenomic tRNAs were most frequent on the anticodon arm (5 mutations), followed by the acceptor stem (3 mutations), and the TψC arm (2 mutations). tRNAs are important nexus molecules between mRNAs and proteins, and they play a central role in translation^42,43^. tRNA mutations can affect the efficiency of protein synthesis, the stress response, and the occurrence of disease^44,45^. However, few studies of tRNA mutations in macro fungi are yet available. Further studies are needed to determine the effects of tRNA mutations on the *L. sulphureus* mitogenome, and how these mutations affect fungal growth and development.

Repetitive elements within the fungal mitogenome contribute to dynamic changes in mitochondrial structure^46^. These changes could influence gene order, lead to the over-dispersal of repeat sequences, or introduce new genes through horizontal gene transfer^47^. In addition, the accumulation of repetitive elements in the mitochondrial genome is associated with increased gene recombination and loss^47^. In the mitochondrial genome of *L. sulphureus*, we identified 36 repeat regions and 15 tandem repeats, accounting for 2.14% and 0.65%, respectively, of the total genome length. The repeats accumulated in the *L. sulphureus* mitogenome might result in variations in gene content and structure, and might promote species differentiation. In addition, the probable transfer of gene fragments between the nuclear and mitochondrial genomes might also be important for species differentiation and evolution. The phenomenon of gene transfer has been observed in many organisms^33,48^. Here, 6.1 kb were aligned between the nuclear and the mitochondrial genomes (a maximum individual alignment length of 654 bp), suggesting that gene transfer had occurred during the evolution of *L. sulphureus*. Further studies are needed to confirm the effects that these gene transfers have had on mitochondrial metabolism.

All mitochondrial genomes derive from a common ancestor, the alpha*-*proteobacteria^18,19^. It is thus remarkable that mitogenomes differ so markedly among different eukaryotic species^47^. Previously, mitochondrial gene order was thought to be relatively conserved^47^, except in plants, where the order of mitochondrial genes was known to vary because of the high rate of recombination^49^. However, as the number of available mitogenomes has increased, so have reports of variations in mirochondrial gene order^50^. The mitogenomes of fungi are less well studied than those of other eukaryotes. Previous studies have shown that fungal mitogenomic gene order is highly variable^51^, primarily because of the accumulation of repetitive sequence in the intergenic regions, in introns, and in intronic ORFs^47^. Here, the mitochondrial gene order varied at different taxonomic levels. In *Ganoderma*, genes were highly syntenic. At the family level, however, the mitogenetic gene order among different genera varied. The arrangement of genes in the mitogenome may be used to assess the phylogenetic relationships among different species.

The 14 conserved PCGs in the fungal mitogenome code for proteins important for energy metabolism; these proteins maintain most of the fungal energy supply. However, several variations in PCGs were found among different *Polyporales* species. We found that, in the *L. sulphureus* mitogenome, the length of 10 PCGs was unique across all seven *Polyporales* species. In addition, the GC skew of most PCGs from most species studied was negative, unlike in the *L. sulphureus* mitogenome. The GC content of most of the 14 conserved PCGs in the *L. sulphureus* mitogenome was higher than that of other *Polyporales* fungi, placing the *L. sulphureus* mitogenome among the most GC-rich of all previously published *Basidiomycetes* mitogenomes. This suggested that the *L. sulphureus* mitogenome either evolved to be GC rich, or inherited GC-richness from an ancestor. The K2P distances among the 14 conserved PCGs and *rps3* ranged from 0.1 to 0.45. The translation protein rps3 and the energy metabolism protein *nad3* had the largest K2P values across all species tested, indicating that these are more greatly differentiated. The Ka/Ks values of the 15 PCGs were <1, indicating that these PCGs were subjected to purifying selection^52,53^.

Mitogenomic analysis has been widely used in evolution and population genetics^54–56^. *L. sulphureus* is morphologically cryptic: there is no effective way to distinguish subspecies or closely related species based on the limited morphological features. Therefore, mitogenomic sequences might be useful tools for accurate species identification^57^. In comparison to species identifications based on phylogenetic analyses of single gene sequences, such as* ITS or* rRNA genes^14,15^, phylogenies based on combined mitochondrial genes are more reliable, because they include more genetic information^58,59^. Here, we inferred the phylogenetic relationships of 18 species of fungi based on 15 mitogenomic PCGs; this analysis recovered a well-supported tree topology divided into three main clusters, consistent with traditional morphological classifications. Phylogenetic analyses based on different single genes recovered varied tree topologies, consistent with previous studies^53^, possibly because single genes might not provide adequate phylogenetic information. In addition, the phylogenetic inference method used and the effects of incomplete taxon sampling should be considered. Nevertheless, the genes *cox1*, *cob*, and *rnl* might be potential molecular markers for future phylogenetic analyses. Our results indicated that the selection of suitable molecular marker sequences is important for phylogenetic and population genetics studies of fungi.

## Materials and Methods

### Strains and DNA extraction

We obtained the mycelia of *L. sulphureus* from the China General Microbiological Culture Collection Center (Beijing; strain number: 5.622). The mycelia were cultivated in liquid potato dextrose medium for five days and then collected for DNA extraction. Genomic DNA was extracted from the mycelia using a fungal DNA kit (Cat. #D3390-00, Omega Bio-Tek, Norcross, GA, USA), following the manufacturer’s instructions.

### Sequencing, assembly and annotation of mitogenome

With the extracted genomic DNA, we constructed sequencing libraries using a NEBNext Ultra II DNA Library Prep Kit (NEB, Beijing, China), following the manufacturer’s instructions. Whole genomic sequencing was performed using an Illumina HiSeq 2500 Platform (Illumina, San Diego, CA, USA). We performed quality control on the sequences and assembled the mitogenome *de novo* following the methods of Kanzi *et al*.^39^. Briefly, the obtained clean reads were screened with bowtie2^60^ using mitogenomes of closely related species as references. We then assembled the mitogenome *de novo* using SPAdes 3.9.0^61^. Gaps between contigs were filled with MITObim 1.9^62^.

The complete mitochondrial genome of *L. sulphureus* was initially annotated with MFannot^63^ and MITOS^64^, based on genetic code 4. Genes initially annotated as protein coding (PCGs), rRNA, or tRNA were then modified by comparison with the previously published mitochondrial genomes in *Polyporales*. We also predicted PCGs of *L. sulphureus* with the NCBI ORF Finder (https://www.ncbi.nlm.nih.gov/orffinder), and annotated the NCBI-predicted PCGs with BLASTp, searching against the NCBI non-redundant protein database^65^. tRNA genes were also predicted with tRNAscan-SE 1.3.1^66^. The graphical map of the complete mitogenome was drawn with GenomeVx (http://wolfe.ucd.ie/GenomeVx/).

### Analysis of mitogenomic organization

We determined the base composition of the mitogenome with Lasergene v7.1 (DNASTAR; http://www.dnastar.com/). We calculated AT skew as (A − T)/(A + T) and GC skew as (G − C)/(G + C), following the methods of Wang *et al*.^53^. For each of the PCGs in the seven previously published *Polyporales* mitogenomes, the synonymous substitution rate (Ks) and the nonsynonymous substitution rate (Ka) were calculated with DnaSP v6.10.01^67^. We calculated the genetic distances for 14 conserved core protein-encoding genes (*atp6*, *atp8*, *atp9*, *cox1*, *cox2*, *cox3*, *nad1*, *nad2*, *nad3*, *nad4*, *nad4L*, *nad5*, *nad6*, and *cob*) and one ribosomal protein S3 gene (*rps3*) with MEGA v6.06 using the Kimura-2-parameter model (K2P)^68^. We also calculated the number and frequency of each codon type with the Codon Usage module of the Sequence Manipulation Suite^69^ using genetic code 4. We compared gene rearrangement and mitogenomic sequence across the previously published *Polyporales* species with Mauve 2.4.0^70^.

### Repetitive elements analysis

Local BLAST searches were performed against the previously published *L. sulphureus* nuclear genome^71^ (GenBank accession number: LOAX00000000.1) to identify gene segments that may have been transferred between the mitochondrial and the nuclear genomes of *L. sulphureus*. To determine whether there had been intra-genomic duplication of large fragments and whether interspersed repeats appeared in the mitogenome of *L. sulphureus*, BLASTn searches of the entire mitogenome against itself were performed with Circoletto^72^ (http://tools.bat.infspire.org/circoletto/) using an E-value of <10^−10^. We also analyzed tandem repeats with the Tandem Repeats Finder^73^ (http://tandem.bu.edu/trf/trf.advanced.submit.html), using default parameters. We searched for repeated sequences in the *L. sulphureus* mitogenome with REPuter^74^, which identifies forward (direct), reverse, complemented, and palindromic (revere complemented) repeats. We used the default settings of REPuter, which filtered repeats with E-values <10^−5^.

### Phylogenetic analysis

To investigate the phylogenetic relationships among *Agaricomycetes* species, we analyzed *L. sulphurus*, the seven *Polyporales* species previously discussed, seven additional *Agaricales* species, two *Cantharellales* species and one *Pucciniomycotina* species as the outgroup. We first aligned the complete nucleotide sequences of 14 conserved core protein-encoding genes individually with MAFFT v7.037^75^. Individual gene alignments were concatenated to create a combined matrix with SequenceMatrix 1.7.8. Modelgenerator v851^76^ was used to determine the best-fit evolutionary model for the combined gene alignment.

We used maximum likelihood (ML) and Bayesian inference (BI) to create phylogenies based on the combined gene alignment. The ML analysis was performed with RAxML^77^; bootstrap values were calculated using 1,000 replicates to assess node support. Bayesian analyses were performed with MrBayes v3.2.6^78^. Two independent runs with four chains each (three hot and one cold) were run simultaneously for 2*10^6^ generations. Each run was sampled every 100 generations. We assumed that the analysis had reached stationarity when the ESS (estimated sample size) value was >100 and the PSRF (potential scale reduction factor) value approached 1.0. The first 25% samples were discarded as burn-in, and the remaining trees were used to calculate posterior probabilities in a 50% majority-rule consensus tree. We also used BI to derive phylogenies for single mitochondrial genes (*atp6, atp8, atp9, cox1, cox2, cox3, nad1, nad2, nad3, nad4, nad4L, nad5, nad6, cob, rps3, rnl*, and *rns*), to evaluate whether these individual genes could be used as molecular markers for phylogenetic analyses of *Agaricomycetes* species.

### Data availability statement

The *L. sulphureus* mitochondrial genome sequence was submitted to GenBank, accession number MG519331. Mitogenomes used for comparative mitogenomic analysis were downloaded from GenBank.

## Electronic supplementary material

Supplementary figure

Supplementary tables
