# Supplementary material for: Characterization and phylogenetic analysis of the complete mitochondrial genome of the medicinal fungus Laetiporus sulphureus
Source: Sci Rep. 2018 Jun 14;8:9104. doi: 10.1038/s41598-018-27489-9 (PMC6002367; doi:10.1038/s41598-018-27489-9)
Supplement: Supplementary file 1 — Supplementary figure [file 41598_2018_27489_MOESM1_ESM.pdf]

Scientific Reports

Characterization and phylogenetic analysis of the complete mitochondrial genome of the medicinal fung us *Laetiporus sulphureus*

Running title: Complete mitogenome of *Laetiporus sulphureus*

Qiang Li[1,3], Mei Yang[2#], Cheng Chen[4#], Chuan Xiong[1], Xin Jin[1], Zhigang Pu[1]*, Wenli Huang[1]*

1. Biotechnology and Nuclear Technology Research Institute, Sichuan Academy of Agricultural Sciences, Chengdu 610061, Sichuan, P.R.China
2. Panzhihua City Academy of Agricultural and Forest Sciences , Panzhihua 617061, Sichuan, P.R.China

3. Key Laboratory of Bio -Resource and Eco -Environment of Ministry of Education, College of Life Sciences, Sichuan University, Chengdu 610065, Sichuan, P.R.China

4. Institute of plant protection, Sichuan Academy of Agricultural Sciences, Chengdu 610066, Sichuan, P.R.China

*Corresponding author:

Zhigang Pu (zhigangpu@126.com) and Wenli Huang (wenlih11@126.com)

Phone: +86-028-84592187;

*Present address: Sichuan Academy of Agricultural Sciences, 106 # Shizishan Rd, Chengdu 610061, Sichuan, China.

# Qiang Li, Mei Yang, and Cheng Chen contributed equally to this work

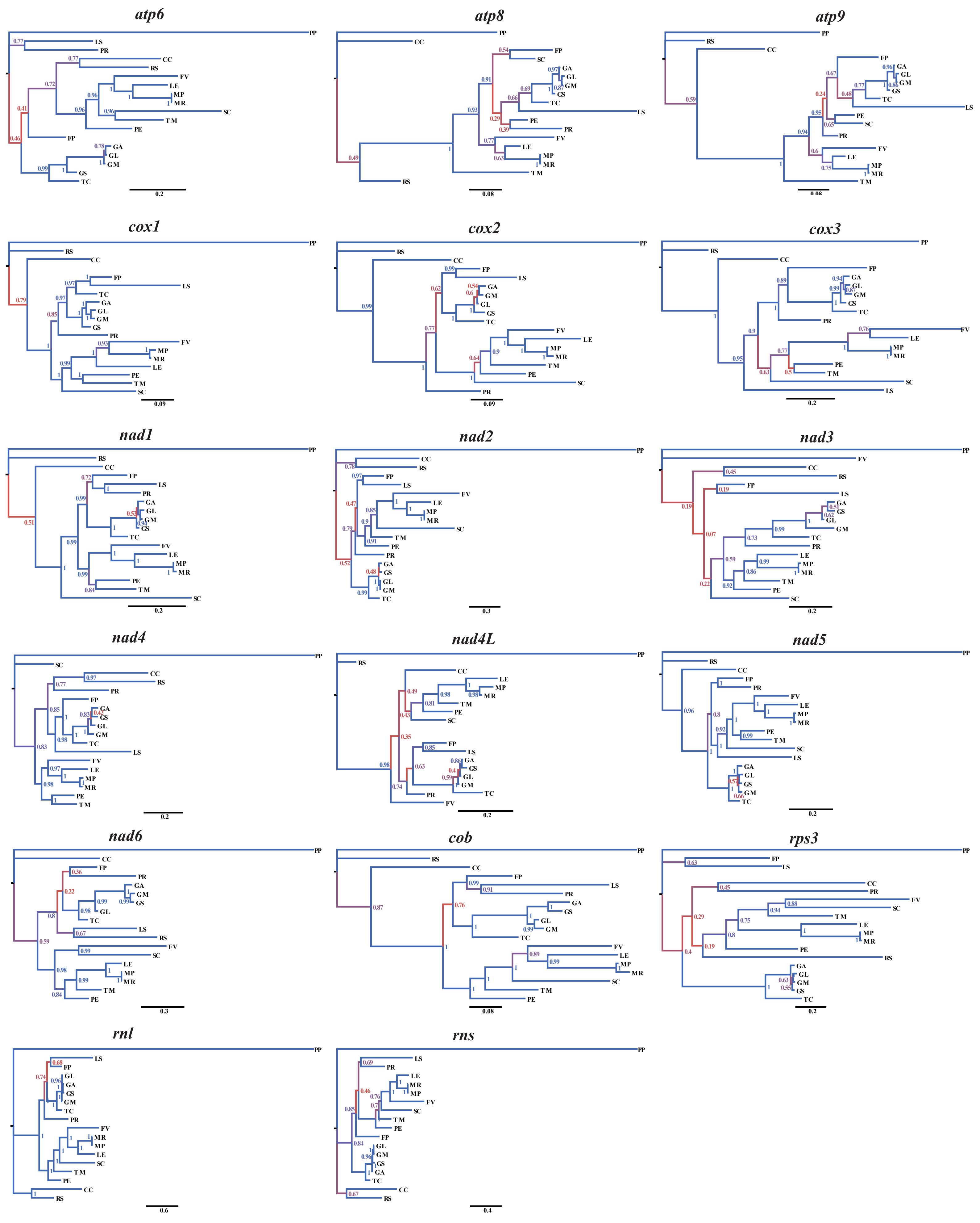

Fig. S1 Bayesian phylogeny of 18 species from *Agaricomycetes* using each of 15 protein-coding genes, *rnL*, and *rnS* gene. Strains used in this study: CC, *Cantharellus cibarius* (NC_020368); FV, *Flammulina velutipes* (NC_021373); FP, *Fomitopsis palustris* (NC_034349); GA, *Ganoderma applanatum* (NC_027188); GL, *Ganoderma lucidum* (NC_021750); GM, *Ganoderma meredithae* (NC_026782); GS, *Ganoderma sinense* (NC_022933); HC, *Hericium coralloides* (NC_033903); LE, *Lentinula edodes* (NC_018365); MP, *Moniliophthora perniciosa* (NC_005927); MR, *Moniliophthora roreri* (NC_015400); PR, *Phlebia radiata* (NC_020148); PE, *Pleurotus eryngii* (NC_033533); RS, *Rhizoctonia solani* (NC_021436); SC, *Schizophyllum commune* (NC_003049); TC, *Trametes cingulate* (NC_013933); TM, *Tricholoma matsutake* (NC_028135); **LS, *Laetiporus sulphureus***; outgroup PP, *Phakopsora pachyrhizi* (NC_014344).
